# P22 Review of antibiotic therapy (lymecycline) for the treatment of acne vulgaris in primary care

**DOI:** 10.1093/jacamr/dlaf118.029

**Published:** 2025-07-14

**Authors:** Adam Young

**Affiliations:** Royal Cornwall Hospital Trust and PCN, Truro, UK

## Abstract

**Background:**

Acne vulgaris is a common dermatological condition affecting a large portion of the population particularly adolescents and young adults. Lymecycline, a tetracycline antibiotic is regularly prescribed in primary care for its treatment. The prescribing of lymecycline, an antibiotic for the treatment of acne vulgaris should be done in accordance with NICE guidelines. Cornwall has amongst the highest prescribing in England with over half of patients receiving treatment for more than 24 weeks, increasing the risk of antimicrobial resistance.^1^

**Objectives:**

Relevant NICE guidance states that patients should be on concurrent topical treatment which is not a topical antibiotic alongside an oral antibiotic (Lymecycline). They should also have 3-month and 6-month reviews to review response to treatment. For women of child-bearing age it is also recommended to be on/ have been offered an effective form of contraception due to the teratogenic effects of tetracycline antibiotics. It also highlights when referral to a specialist should be made/ considered where appropriate. _(1)_ This audit aims to evaluate the surgery’s current prescribing against these NICE criteria when treating and managing acne vulgaris.

**Methods:**

This is a retrospective review of patient records from a GP surgery in Cornwall, focusing on those diagnosed with acne vulgaris and prescribed an oral antibiotic (lymecycline, minocycline, doxycycline, oxytetracycline and tetracycline) over 4 months in Winter 2024. Data collected included: patient demographics (age, gender), specific antibiotic prescribed, duration of therapy, completion of 3- and 6-month reviews, concurrent topical treatments, contraception use and if there was specialist involvement in the patients care and management. Additionally, compliance with clinical guidelines for antibiotic use in acne treatment was assessed.

**Results:**

The records of 39 patients were reviewed to assess the adherence to NICE guidelines. Out of the 39 eligible patients, 21 (53.8%) were not prescribed concurrent topical treatment alongside their oral antibiotics. Seventeen (56.7%) of 30 eligible female patients were not prescribed or shown to be offered a form of effective contraception. Regarding the review of the oral antibiotic treatment, 29 (74.4%) of 39 patients had not had their 3-month review with 33 (84.6%) of 39 also not having had their 6-month review, despite still being prescribed regular courses of lymecycline. There was an overall low compliance with the NICE guidelines.

**Conclusions:**

This audit found significant deviations from the NICE guidelines for acne treatment prescribing. With a substantial proportion of patients not receiving concurrent topical treatments, many women of child-bearing age were not offered effective contraception and majority of patients not undergoing the recommended 3-month and 6-month reviews. Enhanced training for prescribers on appropriate prescribing practices including contraception and systematic review processes, as to limit prescribing to 3-months before prompting a review, may help to address these issues and improve compliance with the guideline standards.
